# Editorial: Rising stars in gastroenterology: 2023

**DOI:** 10.3389/fgstr.2024.1457966

**Published:** 2024-09-27

**Authors:** Endrit Shahini, Deyu Zhang, Jiaoyu Ai, Emanuele Sinagra

**Affiliations:** ^1^ Gastroenterology Unit, National Institute of Gastroenterology-IRCCS “Saverio de Bellis”, Bari, Italy; ^2^ Changhai Hospital, Second Military Medical University, Shanghai, China; ^3^ Department of Gastroenterology, First Affiliated Hospital of Nanchang University, Nanchang, China; ^4^ Gastroenterology and Endoscopy Unit, Fondazione Istituto G. Giglio, Cefalù, Italy

**Keywords:** cryptogenic chronic hepatitis, intrahepatic cholangiocarcinoma, TACE, TARE, radiological interventions for HCC, ultrasound-guided portosystemic pressure gradient, cholangiopancreatoscopy

In this editorial, we will discuss the clinical management of cryptogenic chronic hepatitis, current trends in radiological interventions for intrahepatic cholangiocarcinoma, and technological advancements in managing gastrointestinal, biliary, and pancreatic diseases.

## Hepatology - clues to resolving cryptogenic chronic hepatitis and contemporary trends in radiological interventions for intrahepatic cholangiocarcinoma

Cryptogenic chronic hepatitis affects 5%-15% of patients with chronic liver disease and is an increasing cause of liver transplantation (1). Despite advances in technology, developing countries face difficulties in enhancing diagnostic abilities. In a retrospective study, Cancado et al. re-evaluated clinical, laboratory, imaging, and histological data from 326 patients in Brazil to identify underlying causes of cryptogenic liver disease.

A new algorithm, which included the concept of metabolic-dysfunction-associated fatty liver disease (MAFLD) and excluded lysosomal acid lipase deficiency (LAL-D), allowed for a higher etiological diagnosis in 49.1% of patients compared to 37.2% using the previous Czaja algorithm, resulting in 11.4% fewer liver biopsy cases.

Cocozza et al. conducted a systematic review of 19 TARE and 15 TACE studies for the treatment of intrahepatic cholangiocarcinoma (ICC), which are intra-arterial therapies recommended by current guidelines for patients with non-metastatic disease unfit for surgery. This study confirms previous research, indicating that TACE and TARE have similar overall survival and response rates, with TARE having fewer adverse events. Identifying eligible patients remains a significant challenge due to various populations and small single-centre studies, which result in selection bias and significant heterogeneity. Current evidence favours TACE for patients with a higher performance status or who have not previously received treatment for downstaging to surgery. TARE is the preferred technique for recurrent tumours, whereas the TARE combination is especially appealing because of the complementary effects of chemotherapeutic agents and radiation. Finally, randomised controlled trials (RCTs) are warranted to determine whether TACE is superior to TARE or vice versa.

## Endoscopy - the intersection of technology with endoscopy and surgery: advancements in gastrointestinal, biliary and pancreatic disease management

The endoscopic ultrasound-guided portosystemic pressure gradient (EUS-PPG) measurement procedure allows for direct measurement of the portal pressure gradient as well as simultaneous EUS-guided liver biopsy, evaluation of oesophageal or gastric varices, and portal hypertensive gastropathy, eliminating the need for a separate esophagogastroduodenoscopy (2).

According to a systematic review by Malik et al. including four studies, EUS-PPG technical success rate (under moderate sedation or general anaesthesia) is comparable to interventional radiologist-guided transjugular hepatic venous pressure gradient (IR-HVPG), but with fewer adverse events related to contrast medium. However, additional RCTs and multicentre prospective studies are needed to establish patient selection criteria for EUS-PPG measurement over conventional HVPG and to determine whether there is a consistent correlation between EUS-PPG measurements and histologic staging of liver fibrosis.

Furthermore, for patients with pancreatic lesions, diagnostic procedures such as EUS with fine needle biopsy (EUS-FNB) can cause disproportionate fear and anxiety about receiving an oncological diagnosis (3). From the perspective that what is known is less frightening, there is an increasing interest in using graphic novels for a variety of diseases to improve patient outcomes because it creates an environment in which healthcare providers can successfully interact with patients (4).

In an original prospective pilot study protocol, Rizzo et al. will investigate the impact of colourful graphic novels on stress levels and behavioural responses of patients diagnosed with pancreatic lesions, as well as schedule a EUS-FNB. The authors designed a comic panel with six exciting vignettes that depicted the standard echoendoscopic procedure. Following hospitalisation, patients will be randomly assigned to either the test or control groups. Following the EUS-FNB, all enrolled patients will complete the Beck Anxiety Inventory (BAI) (to assess anxiety severity) and a modified version of the Depression Anxiety Stress Scales-21 (mDASS-21), which does not include the depression subscale. The findings of the pilot study could help guide future clinical trials evaluating graphic novels for anxiety and stress management before EUS procedures.

Spring-mediated distraction enterogenesis (SMDE) is a surgical procedure that uses an intraluminal self-expanding spring to generate mechanical force, resulting in intestinal stretching/sustained axial growth in animal models for severe short bowel syndrome (SBS) patients to prevent chronic intestinal failure. Bautista et al. investigated in a large animal model juvenile mini-Yucatan pigs who had 75% of their small intestine resected with a jejunoileostomy and an intraluminal spring implanted after a one-month adaptation period.

Two months following surgery, the SMDE segments enhanced these adaptive changes. The initial growth observed within the muscularis and serosal layers reversed during the second month, with the mucosal muscularis layer decreasing by 31%, the circular layer decreasing by 50%, the longitudinal muscularis decreasing by 33%, and the serosal layer decreasing by 51%. Similarly, crypt depth increased by 27%, while villus length decreased by 18%. Within 60 days of resection, the muscularis and serosal layers have reversed the initial adaptive processes.

Non-stretched jejunal segments proximal to spring placement and SMDE segments, mucosal muscularis increased by 288% and 169%, compared to the control. The circular layer grew by 105%, the longitudinal layer by 78% and 19%, and the serosal layer by 205% and 49%, respectively.

SMDE improved morphological responses to resection while preventing morphologic attenuation caused by prolonged adaptation. As a result, this novel technique could be a viable alternative to traditional drugs or surgical lengthening procedures, both of which have a high failure rate. The following studies will assess the restoration of functional and absorptive capacities in the extended intestine and the ability to reverse the length achieved after spring removal.

Contemporary medical and surgical treatments for gastrointestinal (GI) defects are ineffective and cause complications, emphasising the need for novel therapeutic approaches. Tissue engineering and regenerative medicine are rapidly evolving fields that combine cell biology, materials science, and physiology to create functional substitutes for damaged or deficient tissue through *de novo* organogenesis (5). Liu et al.‘s review examines recent advances in GI tract tissue engineering, with a focus on technologies with clinical translational potential between GI surgery and regenerative medicine. In this regard, autologous cell sheets and decellularised scaffolds can successfully repair partial and full-thickness oesophageal defects (such as postendoscopic submucosal dissection, perforations, leaks, and fistulas) in animals and humans. A few studies have shown that autologous pluripotent cells and scaffolds can repair even long-segment circumferential defects in the oesophagus. Animal studies have shown that multipotent cells and scaffolds can repair partial and full-thickness stomach defects. Pluripotent cells and scaffolds may be used to repair partial and full-thickness intestinal defects. Multiple human trials have shown that mesenchymal stem cell therapies, with or without bioscaffolds, are effective in treating full-thickness defects in the rectum and anus, including perianal fistulas, whose surgical treatment may have a low success rate and a high risk of complications, particularly in refractory and challenging cases. However, the optimal production (scaling up manufacturing and cost) and the biomechanics compatibility, integration, and safety of grafts continue to be barriers to clinical translation. Future prospective studies in large animal models and humans will probably more accurately estimate the grafts’ *in-vivo* safety and physiological functions.

Gopakumar and Sharma‘s narrative review discusses the various approaches to peroral cholangiopancreatoscopy, and its established and emerging diagnostic and therapeutic indications. They also discuss the current limitations and potential future applications of cholangioscopy and pancreatoscopy in treating various biliary and pancreatic pathologies.

To date, cholangioscopy should be considered essential in diagnosing indeterminate biliary strictures, as the procedure is associated with high procedural success in terms of diagnostic accuracy, changes clinical outcome in more than 80% of considered insolvable cases, and has an acceptable safety profile (6). Furthermore, cholangioscopy, combined with Narrow-band Imaging (NBI), can be an excellent tool for assessing indeterminate biliary strictures. Cholangioscopy-assisted treatments are highly effective and safe for difficult biliary stones, even in patients whose previous conventional endoscopies to treat biliary stones have failed (7).

The majority of data on RFA and PDT for biliopancreatic malignancies in the context of cholangiocarcinoma treatment come from ERCP-guided therapy. However, the results of recent limited studies on the role of cholangioscopy-directed RFA and PDT are promising, although more extensive research, including head-to-head trials, is required to evaluate their potential superiority in treating unresectable cholangiocarcinoma.

Also, cholangioscopy-directed lithotripsy for complex and symptomatic pancreatolithiasis could be a promising addition to the current endoscopic treatment options. Several studies have shown that image-enhancing technologies such as NBI and confocal laser endomicroscopy (pCLE) improve the diagnostic accuracy of peroral pancreatoscopy in evaluating pancreatic strictures (8, 9).

In this era of rapid technological advancement, the authors believe that as the cholangioscope is developed and more clinical experience is gained its applications will expand even further.

## References

[1] Kodali VP Gordon SC Silverman AL McCray DG. Cryptogenic liver disease in the United States: further evidence for non-A, non-B, and non-C hepatitis. Am J Gastroenterol. (1994) 89:1836–9. 7942678

[2] Lesmana CRA. Endoscopic ultrasound-guided portal pressure gradient measurement in managing portal hypertension. World J gastrointestinal Surg. (2023) 15:1033–9. doi: 10.4240/wjgs.v15.i6.1033 PMC10315130 37405096

[3] Semenenko E Banerjee S Olver I Ashinze P. Review of psychological interventions in patients with cancer. Support Care Cancer Off J Multinatl Assoc Support Care Cancer. (2023) 31:210. doi: 10.1007/s00520-023-07675-w 36913136

[4] De Stefano A Rusciano I Moretti V Scavarda A Green MJ Wall S. Graphic medicine meets human anatomy: The potential role of comics in raising whole body donation awareness in Italy and beyond. A pilot study. Anat Sci Educ. (2023) 16:209–23. doi: 10.1002/ase.2232 36346170

[5] Dua KS Hogan WJ Aadam AA Gasparri M. *In-vivo* oesophageal regeneration in a human being by use of a non-biological scaffold and extracellular matrix. Lancet. (2016) 388:55–61. doi: 10.1016/S0140-6736(15)01036-3 27068836

[6] Pallio S Sinagra E Santagati A D’Amore F Pompei G Conoscenti G. Use of catheter-based cholangioscopy in the diagnosis of indeterminate stenosis: a multicenter experience. Minerva Gastroenterol (Torino). (2024) 70:29–35. doi: 10.23736/S2724-5985.22.02889-3 35262304

[7] Pallio S Sinagra E Santagati A D’Amore F Rossi F Conoscenti G. Digital single-operator cholangioscopy in treating difficult biliary stones: results from a multicenter experience. Minerva Gastroenterol (Torino). (2023) 69:261–7. doi: 10.23736/S2724-5985.21.02892-8 34240591

[8] Miura T Igarashi Y Okano N Miki K Okubo Y. Endoscopic diagnosis of intraductal papillary-mucinous neoplasm of the pancreas by means of peroral pancreatoscopy using a small-diameter videoscope and narrow-band imaging. Dig Endosc. (2010) 22:119–23. doi: 10.1111/j.1443-1661.2010.00926.x 20447205

[9] Itoi T Sofuni A Itokawa F Kurihara T Tsuchiya T Ishii K. Initial experience of peroral pancreatoscopy combined with narrow-band imaging in the diagnosis of intraductal papillary mucinous neoplasms of the pancreas (with videos). Gastrointest Endosc. (2007) 66:793–7. doi: 10.1016/j.gie.2007.03.1096 17905024

